# Quantitative anatomy of the fused ossification center of the occipital squama in the human fetus

**DOI:** 10.1371/journal.pone.0247601

**Published:** 2021-02-23

**Authors:** Magdalena Grzonkowska, Mariusz Baumgart, Mateusz Badura, Marcin Wiśniewski, Michał Szpinda

**Affiliations:** Department of Normal Anatomy, The Ludwik Rydygier Collegium Medicum in Bydgoszcz, The Nicolaus Copernicus University in Toruń, Bydgoszcz, Poland; Institute of Evolutionary Biology, Pompeu Fabra University, SPAIN

## Abstract

CT-based quantitative analysis of any ossification center in the cranium has not previously been carried out due to the limited availability of human fetal material. Detailed morphometric data on the development of ossification centers in the human fetus may be useful in the early detection of congenital defects. Ossification disorders in the cranium are associated with either a delayed development of ossification centers or their mineralization. These aberrations may result in the formation of accessory skull bones that differ in shape and size, and the incidence of which may be misdiagnosed as, e.g., skull fractures. The study material comprised 37 human fetuses of both sexes (16♂, 21♀) aged 18–30 weeks. Using CT, digital image analysis software, 3D reconstruction and statistical methods, the linear, planar and spatial dimensions of the occipital squama ossification center were measured. The morphometric characteristics of the fused ossification center of the occipital squama show no right—left differences. In relation to gestational age, the ossification center of the occipital squama grows linearly in its right and left vertical diameters, logarithmically in its transverse diameters of both the interparietal and supraoccipital parts and projection surface area, and according to a quadratic function in its volume. The obtained numerical findings of the occipital squama ossification center may be considered age-specific references of relevance in both the estimation of gestational age and the diagnostic process of congenital defects.

## Introduction

The ossification process in the cranium begins as early as at 6–7 weeks of the embryonic life from the templates of the occipital bone, which comprises the following five components: basilar part, two lateral parts, as well as lower supraoccipital and upper interparietal parts of the squama. Ossification in the cranium is the last and most complex stage of development. Cranial bones develop within both the chondrocranium and the membranous cranium. Except for the upper part of its squama, the occipital bone develops from the primary chondrocranium and belongs to both the vault and the base of the skull [1–5]. The ossification process of the cranial base starts with the occipital bone and gradually progresses toward the sphenoid and ethmoid bones [1, 6, 7].

The basilar part of the occipital bone is formed by the fusion of four primary cranial cartilages, which are subsequently joined with the sphenoid’s body via the spheno-occipital synchondrosis and contribute to the formation of the clivus. Ossification of the basilar part begins at week 9 of gestational age from a single ossification center. The process evolves laterally toward the lateral parts of the sphenoid bone [1, 4]. Furthermore, according to Jeffery and Spoor [7], ossification of the basilar part of the occipital bone commences as late as week 12 of gestational age.

The occipital squama consists of two parts: lower supraoccipital and upper interparietal ones. Its supraoccipital part is located between the posterior edge of the foramen magnum and the supreme nuchal line. Its interparietal part, on the other hand, covers the area between the supreme nuchal line and the parietal bones [2]. The ossification process of the lower part of the squama originates at weeks 6–7 of the embryonic life from ossification centers that aggregate into one at approximately 9–10 weeks of fetal age. The upper part of the squama ossifies from two ossification centers that fuse into a single center, as with its lower part. The fusion of the ossification centers of the upper and lower parts in the central region occurs at approximately 12 weeks of gestational age [3, 4]. The ossification centers of the occipital squama develop upwards and sideways to form the lateral parts of the squama and to join the opposite side in the central part [2]. The lateral parts of the squama have a wedge-shaped bone-free area that gradually decreases in size, forming a cleft in the newborn [3, 4].

**Fig 1:**
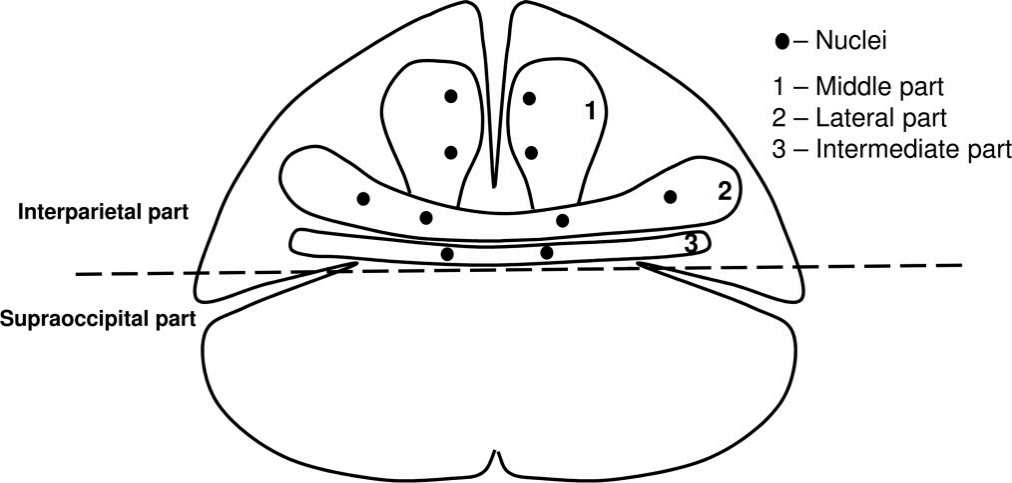
The development of the occipital squama.

However, the development of the occipital squama still remains debatable. According to Srivastava [8], fetuses at 9 weeks of gestational age have two ossification centers at level with the occipital protuberance, located on either side of the posterior midline of the body. When these centers have fused, the intermediate part of the squama is formed, i.e. the area between the superior and supreme nuchal lines. Above this area is another pair of ossification centers, each with two nuclei, medial and lateral, from which the lateral parts of the occipital squama develop. Above them are situated two further ossification centers that form the middle part of the squama. The author suggested that the third pair of ossification centers also had two nuclei per center–upper and lower ones. In fetuses between 12 and 15 weeks of gestational age, the intermediate part is already well-developed. It is joined throughout the length with the subjacent part and, in the central region, with the suprajacent part. The lateral parts are separated from the intermediate part by a lateral cleft. The lateral parts of the squama are fused in the mid-sagittal plane, while the middle part is still divided by a deep medial cleft. At 16 weeks of gestational age, two separate components of the middle part fuse, leaving a small part in the lower section of the bone (Fig 1).

Nikolova et al. [2] and Matsumura et al. [9] elucidate this number of ossification centers by the presence of primary and secondary ossification centers. In their opinion, secondary ossification centers are located above the supreme nuchal line.

To date, CT-based quantitative analyses of cranial bone ossification centers have not been carried because of the limited availability of fetal material [10]. Detailed morphometric data on the development of ossification centers in human fetuses can be useful in the early detection of developmental defects. Ossification disorders in the cranium are associated with a delayed development of ossification centers and their mineralization. This aberration can result in the formation of additional skull bones that differ in shape and size, and their occurrence may be misdiagnosed as, e.g., skull fractures [11, 12].

An understanding of the growth dynamics of individual skull bones in the human fetus, including the occipital bone, may be useful in such fields as anatomy, anthropology, surgery, forensic medicine, neurosurgery, obstetrics, pediatrics, orthopedics and radiology [3, 13].

Although the timing of ossification of each bone in the skull is recognized, no morphometric measurements involving the use of CT examinations of the occipital ossification centers have been reported. This is the first report in the literature to analyze morphometric parameters of the occipital squama ossification centers in human fetuses based on computed tomography imaging.

In the present study we aimed:

- to determine normative age-specific values for linear, planar and volumetric parameters of the occipital squama ossification center in human fetuses;
- to compute growth dynamics for the analyzed parameters, expressed by best-matched mathematical models.

## Material and methods

The study material comprised 37 human fetuses (16 males and 21 females) of Caucasian origin aged 18 to 30 weeks of gestation, derived from spontaneous miscarriages and preterm deliveries. The fetuses were collected before the year 2000 and still remain part of the fetal collection of the Department of Anatomy of Ludwik Rydygier Collegium Medicum of Nicolaus Copernicus University in Toruń.

The parents provided informed written consent to have the fetuses used in research. The experiment was approved by the Bioethics Committee of Nicolaus Copernicus University in Toruń (KB 275/2011). The morphometric examinations were carried out between the 1st of January 2020 and 31st of September 2020, at the Department of Anatomy of Ludwik Rydygier Collegium Medicum of Nicolaus Copernicus University in Toruń. The inclusion criteria of the investigated fetuses were based on their macroscopic examination and statistical cards with the course of pregnancy. As a prerequisite, fetuses with conspicuous developmental diseases such as congenital defects, genetic disorders or intrauterine growth retardation were excluded from the study. Since neither internal nor external noticeable morphological malformations were found in direct morphology assessment, all included specimens were identified as normal. Of note, the fetuses did not display any developmental abnormalities of the musculoskeletal system or chromosomal disorders. Gestational age was determined based on the crown–rump length (CRL) and the known date of the beginning of the last maternal menstrual period. Furthermore, the investigated fetuses could not suffer from growth retardation, as the correlation between the gestational age based on the CRL and that calculated by the last menstruation reached R = 0.98 (p < 0.001). Table 1 lists the characteristics of the study group, including age, number and sex of the fetuses.

**Table 1:** Age, number and sex of the fetuses examined.

Gestational age (weeks)	Crown–rump length (mm)				Number of fetuses	Sex	
	Mean	SD	Min.	Max.	N	♂	♀
18	133.33	5.77	130.00	140.00	3	1	2
19	146.50	2.89	143.00	150.00	4	2	2
20	161.00	2.71	159.00	165.00	4	2	2
21	173.67	2.31	171.00	175.00	3	2	1
22	184.67	1.53	183.00	186.00	3	1	2
23	198.67	2.89	197.00	202.00	3	1	2
24	208.00	3.56	205.00	213.00	4	1	3
25	214.00		214.00	214.00	1	0	1
26	229.00	5.66	225.00	233.00	2	1	1
27	240.33	1.15	239.00	241.00	3	3	0
28	249.50	0.71	249.00	250.00	2	0	2
29	253.00	0.00	253.00	253.00	2	0	2
30	262.67	0.58	262.00	263.00	3	2	1
Total					37	16	21

**Fig 2:**
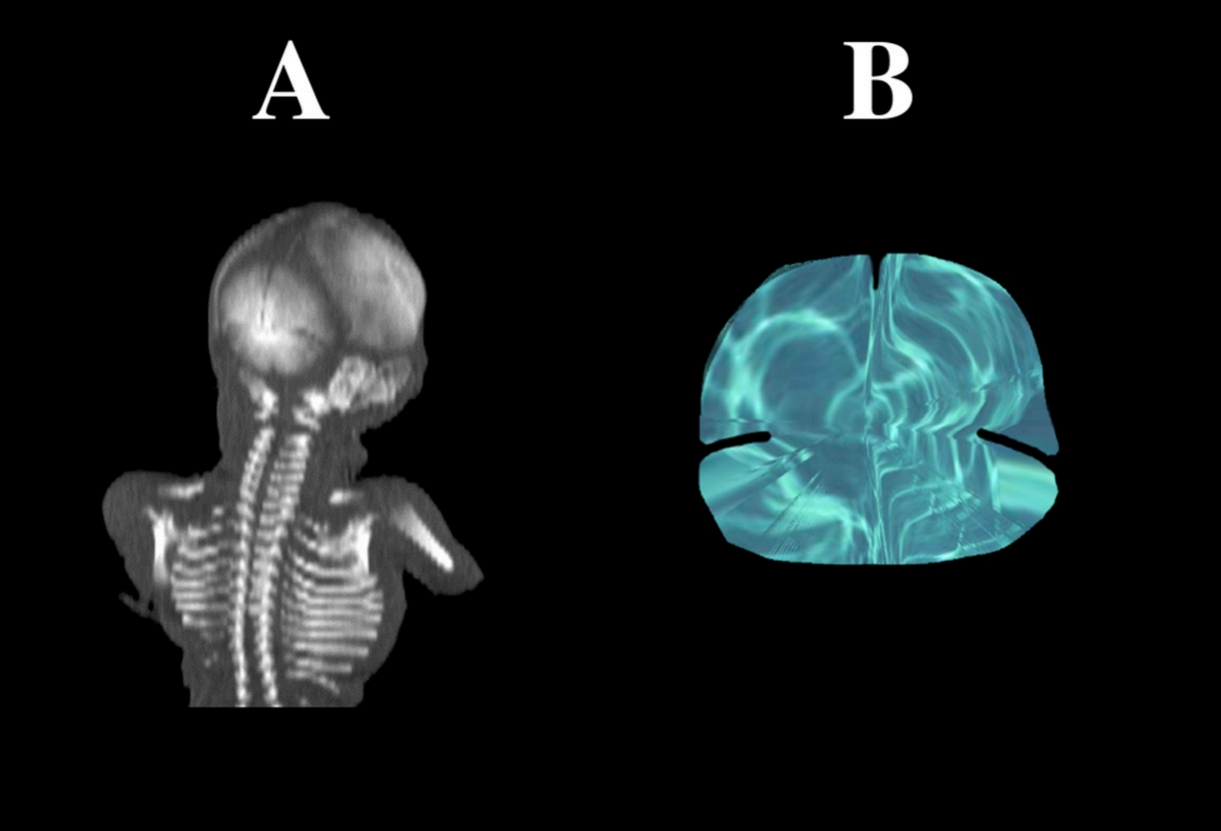
A male human fetus aged 20 weeks on frontal projection (A), 3D reconstruction of the fused occipital squama ossification center (B) using Osirix 3.9 MD. The gray scale of the obtained CT images expressed in Hounsfield units (HU) varied from −275 to −134 for minimum, and from +1165 to +1558 for maximum. Therefore, the window width (WW) was varied from 1.404 to 1.692, and the window level (WL) varied from +463 to +712. Parameters of the imaging protocol were as follows: mAs: 60, kV: 80, pitch: 0.35, FoV: 180, rot. time: 0.5 sec.; while those of the CT data were as follows: slice thickness: 0.4 mm, image increment: 0.6 mm, and kernel: B45 f-medium.

Using the Siemens–Biograph 128 mCT scanner (Siemens Healthcare GmbH, Erlangen, Germany) located at the Department of Positron Emission Tomography and Molecular Imaging (Oncology Center, the Ludwik Rydygier Collegium Medicum in Bydgoszcz, The Nicolaus Copernicus University Bydgoszcz, Poland), scans of fetuses in DICOM formats were acquired at 0.4 mm intervals (Fig 2), and subsequently subjected to morphometric analysis with the use of the Osirix 3.9 MD software. It should be emphasized that Osirix 3.9 MD allows for precise numerical analysis of any linear, planar and three-dimensional reconstructions of objects studied.

Despite the cartilaginous stage of development, a morphometric analysis regarding its vertical and transverse diameters and volume was feasible, as the contours of the entire bone were already evidently visible [14, 15]. In order to precisely visualize and measure the occipital squama ossification center, the resulting fetal scans had to be rotated in relation to the three reference axes: vertical (cranial–caudal), horizontal and sagittal, in order to reach a reference position. It is noteworthy that in this required position, the vertical, horizontal and sagittal axes always traversed the precise center of the occipital squama ossification center, and were set at a right angle in relation to each other. Of note, maintaining these landmarks allowed absolute consistency of measurements. Additionally, such positioning of these three axes allowed setting the occipital squama ossification center accurately in the frontal projection.

**Fig 3:**
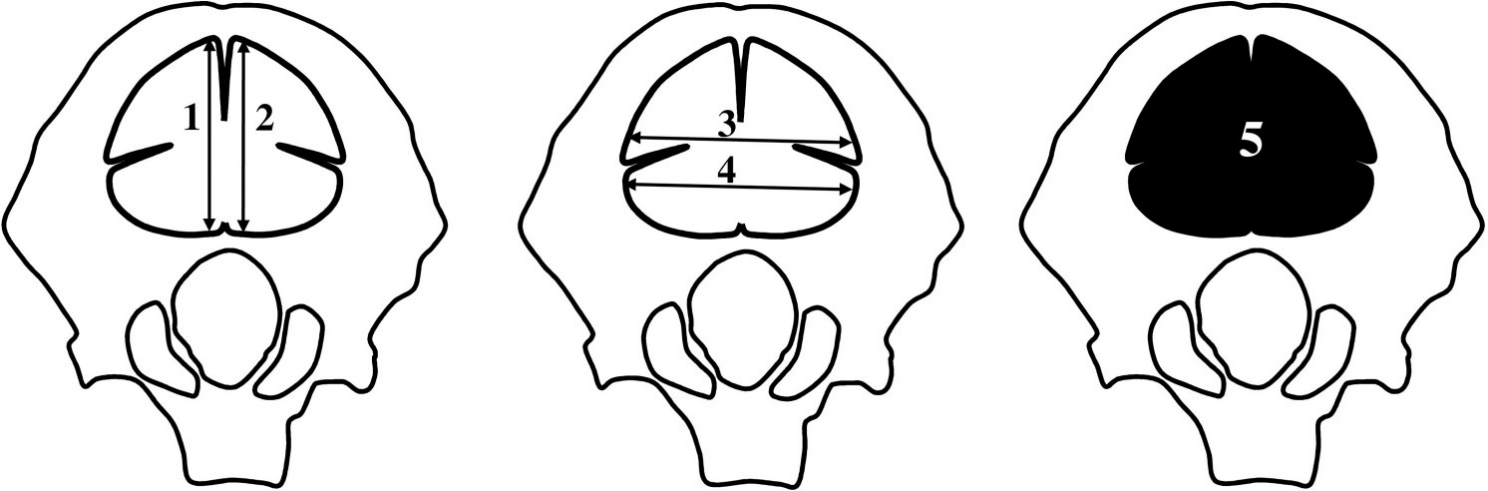
Measurement scheme of the primary ossification center of the occipital squama in the the frontal plane: 1 –right vertical diameter, 2 –left vertical diameter, 3 –transverse diameter of the interparietal part, 4 –transverse diameter of the supraoccipital part, 5 –projection surface area.

Measurements of the occipital squama ossification center (right and left) were performed, as follows (Fig 3):

1. right vertical diameter, based on the determined distance between its proximal and distal borderlines in the frontal plane (Fig 3);
2. left vertical diameter, based on the determined distance between its proximal and distal borderlines in the frontal plane (Fig 3);
3. transverse diameter of the interparietal part, based on the determined distance between its medial and lateral borderlines in the frontal plane (Fig 3);
4. transverse diameter of the supraoccipital part, based on the determined distance between its medial and lateral borderlines in the frontal plane (Fig 3);
5. projection surface area, based on the contoured area involved in the ossification center of the occipital squama in the frontal plane (Fig 3);
6. volume, calculated using advanced diagnostic imaging tools for 3D reconstruction, taking into account position and the absorption of radiation by bone (Fig 2B).

In the present study, the Statistica 12.5 and PQStat 1.6.2. programs were used to statistically analyze all numerical data. Distribution of variables was checked using the Shapiro–Wilk (W) test, while homogeneity of variance was checked using Fisher’s test. To compare the means, Student’s t test for dependent (left–right) variables was used. Afterwards, one-way analysis of variance and Tukey’s test were used for post-hoc analysis. If no similarity of variance occurred, the non-parametric Kruskal–Wallis test was used. The characterization of developmental dynamics of the analyzed parameters was based on linear and curvilinear regression analysis. The match between the estimated curves and measurement results was evaluated on the basis of the coefficient of determination (R^2^). Differences were considered statistically significant at p < 0.05. The relationship between variables was also estimated with the Pearson correlation coefficient (r).

In a continuous effort to minimize measurement and observer bias, all measurements were performed by one researcher (M.B.) and verified by the same examiner. Each measurement was done three times under the same settings but at different times (in one-day intervals), and then averaged. The intra-observer variation between repeated measurements was assessed by ANOVA and post-hoc RIR Tukey test. As exposed in Table 2, the intra-class correlation coefficients (ICC) calculated on the basis of an observer were statistically significant (p < 0.001) and of excellent reproducibility.

**Table 2:** Intra-class correlation coefficient (ICC) values for inter-observer recurrence.

Parameter	ICC
Right vertical diameter	0.997*
Left vertical diameter	0.998*
Transverse diameter of interparietal part	0.999*
Transverse diameter of supraoccipital	0.996*
Projection surface area	0.998*
Volume	0.999*

Intra-class correlation coefficients marked with *are statistically significant at p<0.0001.

## Results

Mean values and standard deviations of the analyzed parameters of the occipital squama ossification centers in human fetuses at the analyzed gestational ages are presented in Tables 3 and 4 for the vertical (right and left) and transverse diameters of the interparietal and supraoccipital parts, projection surface area and volume.

**Table 3:** Right and left vertical and transverse diameters of the interparietal and supraoccipital parts of the fused occipital squama ossification center.

Gestational age (weeks)	Number of fetuses	Fused occipital squama ossification center							
		right vertical diameter (mm)		left vertical diameter (mm)		interparietal part transverse diameter (mm)		supraoccipital part transverse diameter (mm)	
		Mean	SD	Mean	SD	Mean	SD	Mean	SD
18	3	12.68	0.56	12.31	0.03	16.13	1.17	12.42	0.90
19	4	13.95	0.48	13.26	0.94	18.10	0.34	13.94	0.26
20	4	16.04	0.57	15.59	0.52	19.55	0.73	15.05	0.56
21	3	17.47	0.09	17.10	0.29	22.08	0.29	17.30	0.47
22	3	17.84	0.12	17.85	0.35	24.32	1.45	19.22	1.15
23	3	18.41	0.44	18.83	0.36	25.89	0.51	21.75	0.43
24	4	20.18	0.62	21.08	0.82	28.15	1.07	23.64	0.90
25	1	22.17		22.39		30.53		24.73	
26	2	22.82	0.59	23.28	0.06	31.84	0.42	25.42	0.05
27	3	23.55	0.15	24.29	0.59	32.44	0.34	25.66	0.24
28	2	24.24	0.08	25.10	0.06	33.87	0.32	26.70	0.17
29	2	24.92	0.08	25.55	0.08	34.41	0.04	26.90	0.05
30	3	26.30	1.04	27.10	0.70	35.74	0.84	27.88	0.66

**Table 4:** Projection surface area and volume of the fused occipital squama ossification center.

Gestational age (weeks)	Number of fetuses	Fused occipital squama ossification center			
		projection surface area (mm^2^)		volume (mm^3^)	
		Mean	SD	Mean	SD
18	3	175.70	13.12	191.60	16.04
19	4	206.30	9.80	233.71	13.31
20	4	228.25	14.09	269.96	17.80
21	3	282.85	9.73	337.57	13.09
22	3	326.51	23.76	394.08	30.43
23	3	376.75	14.58	458.43	19.60
24	4	458.90	33.77	568.11	45.95
25	1	519.73	-	649.67	-
26	2	559.81	5.92	713.77	11.50
27	3	592.83	19.93	778.71	31.87
28	2	638.37	6.53	871.44	22.46
29	2	657.97	3.01	944.22	18.28
30	3	724.33	31.76	1074.64	54.54

The statistical analysis revealed no laterality differences, which allowed us to compute one growth curve for each analyzed parameter. The developmental dynamics of the right and left vertical diameters followed linear functions, while that of the transverse diameters followed logarithmic functions.

**Fig 4:**
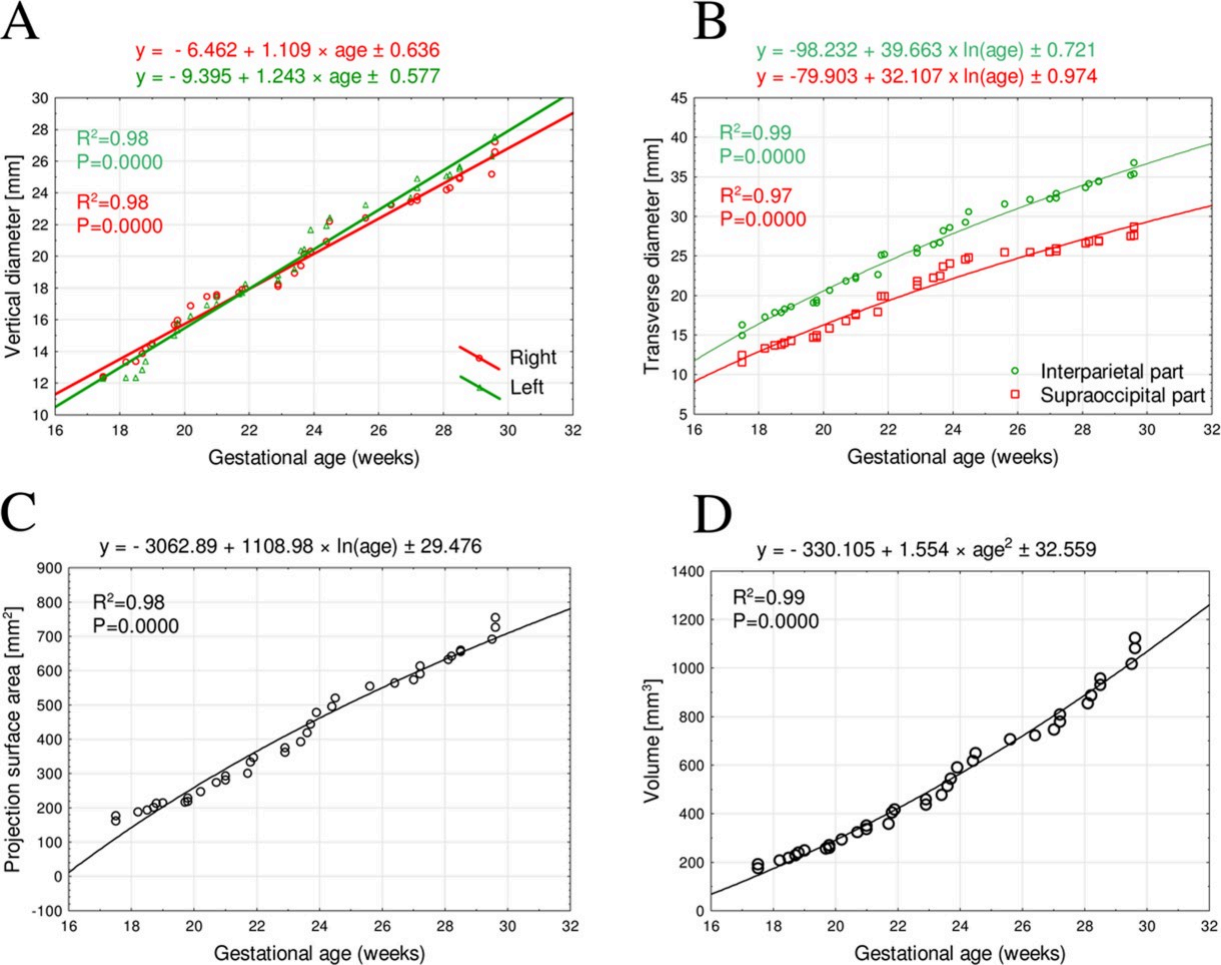
Regression lines for right and left vertical diameters (A), transverse diameters of the interparietal and supraoccipital part (B), projection surface area (C), and volume (D) of the fused occipital squama ossification center. The mean transverse diameter of the interparietal part of the occipital squama ossification center at the gestational ages of 18–30 weeks ranged from 16.13 ± 1.17 to 33.87 ± 0.32 mm, following the logarithmic function: y = -98.232 + 39.663 × ln(age) ± 0.721 R^2^ = 0.99 (Fig 4B), while the transverse diameter of the supraoccipital part ranged from 12.42 ± 0.90mm to 26.70 ± 0.17mm, following the function: y = -79.903 + 32.107 × ln(age) ± 0.974 R^2^ = 0.99 (Fig 4B).

The mean vertical diameter of the occipital squama ossification center at the gestational age range of 18–30 weeks was between 12.68 ± 0.056 mm and 26.30 ± 1.04 mm on the right side, and between 12.31 ± 0.03 mm and 27.10 ± 0.70 mm on the left side, following the linear functions: y = -6.462 + 1.109 × age ± 0.636 R^2^ = 0.98 for the right side (Fig 4A) and y = -9.395 + 1.243 × age ± 0.577 R^2^ = 0.98 for the left side (Fig 4A).

The mean projection surface area of the occipital squama ossification center ranged from 175.70 ± 13.12mm^2^ at 18 weeks to 724.33 ± 31.76mm^2^at 30 weeks of gestational age, following the logarithmic function: y = -3062.89 + 1108.98 × ln(age) ± 29.476 R^2^ = 0.98 (Fig 4C).

The mean volume of the occipital squama ossification center at the gestational age range of 18–30 weeks was between 191.60 ± 16.04 mm^3^and 1074.64 ± 54.54 mm^3^, following the quadratic function of age: y = -330.105 + 1.554 × age^2^ ± 32.559 R^2^ = 0.99 (Fig 4D).

## Discussion

Matsumura et al. [9] examined 752 human fetuses at the age of 3–10 months of gestational age. In 11 fetuses at 3 months of gestational age, the authors found one more ossification center to be present in the supraoccipital part, and in 3 cases an additional third pair of ossification centers was visible. In contrast, in the interparietal part at 3 months of gestational age, a pair of primary ossification centers or one fused primary ossification center could still be observed. At later stages, two pairs of secondary ossification centers appeared, sited above the primary centers. Secondary ossification centers were visible in all fetuses at 4 months of gestational age.

Furthermore, Murlimanju et al. [16] concluded that the upper part of the occipital squama had two ossification centers, and occasionally, even a third center was located in its upper central part.

Mandarim-de-Lacerda and Alves [13] in a study on human fetuses weighed each bone of the skull. They noticed that the facial skeleton bones (vomer, palatine bone, mandible and maxilla) grow in a manner different from that of the neurocranial bones (sphenoid, ethmoid, frontal, occipital, parietal and temporal). The growth of the neurocranial bones was much faster than that of the facial skeleton. Moreover, the fastest growth in the neurocranium was noted for the bones forming the calvaria.

Studies on fetal skulls confirmed that the growth of the skull was faster lengthwise than width- or heightwise [6, 10]. Numerous studies also proved that there were no sex differences in relation to skull shape and ontogenetic trajectory during the prenatal period [10, 17, 18].

From a clinical point of view, the occipital squama ossification center is the most conspicuous ossification center of the occipital bone on routine ultrasound examination. The fusion of all parts of the occipital squama refers to at approximately 12 weeks of gestational age. Our study involves mathematical models that describe the growth of the occipital squama ossification center in terms of its both interparietal and supraoccipital parts. Some disturbances in the growth of the examined parameters after 12 weeks of gestational age–in our study, a period from 18 to 30 weeks–may indicate a possible skeletal dysplasia, such as Chiari malformation, cerebral hernia, and occipital horn syndrome.

This paper is the first report in the professional literature to precisely analyze morphometric parameters of the occipital squama ossification center in human fetuses with mathematical growth models. The right and left vertical diameters of the occipital squama ossification center followed linear functions, therefore grew proportionately to gestational age in weeks, which proved an intense and dynamic growth of this ossification center between 18 and 30 weeks of gestational age. In turn, the transverse diameters of the supraoccipital and interparietal parts, as well as the projection surface area of the occipital squama ossification center followed natural logarithmic functions, with gestational age expressed in weeks. In the studied period, a logarithmic increase of the parameters studied indicates an intensive development: up to 27 weeks of gestation for the interparietal part, up to 26 weeks of gestation for the supraoccipital part, and up to 28 weeks for projection surface area of the occipital squama ossification center. Gradual inhibition of growth in the subsequent weeks of development. Moreover, in the analyzed period, the mean transverse diameter of the interparietal part was 20 percent larger than that of the supraoccipital part. The study also revealed that the volume of the occipital squama ossification center increased in accordance with a quadratic function of gestational age. In a study on the development of the frontal squama ossification center in human fetuses, the growth dynamics regarding its vertical diameter, projection surface area and volume followed the quadratic functions: y = 13.756 + 0.021 × age^2^ ± 0.024, y = 38.285 + 0.889 × age^2^ ± 0.034, and y = -90.756 + 1.375 × age^2^ ± 11.44, respectively. In turn the transverse diameter increased in a manner directly proportionate to gestational age, following the linear function:y = 0.956 + 0.956 × age ± 0.823 [19].

Unfortunately, a lack of numerical data concerning the occipital squama ossification center in the medical literature limits a more detailed discussion on this topic.

The dimensions of the occipital squama ossification center obtained in the present study may be potentially useful in diagnosing skeletal dysplasias that are often characterized by a disrupted or completely halted growth of the occipital bone in the fetus. Our numerical data referring to the occipital squama ossification center may be relevant in monitoring normal fetal growth and screening for congenital disorders. Skeletal dysplasias also involve other bones of the neurocranium (frontal, parietal, and occipital) and their ossification along with development of the neural tube. Disturbances of the ossification process may result in separation of two parts of the occipital squama by the transverse occipital suture at the level of the supreme nuchal line [8, 11] In such cases, the separate interparietal part constitutes a discrete bone called an Inca bone or an interparietal bone [3, 11]. The frequency of occurrence of interparietal bones varies between populations. It is estimated that such bones occur in 15% of Nigerians, 4.8% of the South American population, 2.4% of Indians, 1.2% of Europeans and 0.8% of Australians [3]. Moreover, due to the presence of paired ossification centers in the upper part of the occipital squama, multiple interparietal bones can form spontaneously [1]. Such variations can be misdiagnosed by clinicians or diagnosticians as skull fractures [16].

Occasionally an additional ossification center that appears anteriorly to the interparietal part may not fuse with the rest, thus resulting in the formation of a preinterparietal bone. Interparietal bones may be single, double, triple or even quadruple, with two out of four bones that occur in [20] described a case with a tripartite interparietal bone, while

Gopathan [21] found a 5-part preinterparietal bone in 0.8% of cases. Murlimanju et al. [16], in their study of skulls of the Karnataka population, observed interparietal bones in 3.8% and preinterparietal bones in 10.3% of the studied cases, with both such anomalies observed more frequently in female skulls. These finding are different from those reported by Carolineberry and Berry [22] and by Marathe et al. [23], who observed higher incidences in male skulls. Other typical places with additional bones are observed within the lambdoid suture and the posterior fontanelle, in which separate ossification centers form the so-called Wormian bones [2]. Their excessive number or size may be symptomatic of various pathologies. A feature distinguishing Wormian bones from preinterparietal bones is the irregularity of the shape and position of the former. According to Matsumura et al. [9], Wormian bones in the posterior fontanelle do not take a triangular appearance and do not form a depression in that region. Moreover, Wormian bones are typically sited in narrow spaces between the parietal bones and the interparietal part of the occipital squama, which can be observed after the 6th month of gestational age. In turn, preinterparietal bones form in the 5th month of gestational age. According to Das et al. [24], interparietal bones are often accompanied by a persistent frontal suture and Wormian bones in the posterior fontanelle and lambdoid suture.

Congenital cerebrospinal anomalies are often associated with congenital cranial anomalies, so the analysis of growth of the cranial base bones is relevant from a clinical perspective [6]. The shallowing of the posterior cranial fossa is typical of Chiari malformation, in which structures of the rhombencephalon are displaced into the spinal canal. In this malformation type, parts of the occipital bone can be hypoplastic and underdeveloped. Dysplasias of the occipital bone are non-linear, with its various parts being affected in a disproportionate manner [1]. Other developmental defects of the fetal skull may be caused by cerebral hernia (encephalocele). It is a defect that refers to the 4th week of pregnancy as a result of, e.g., maternal deficiency of folic acid, genetic defects or viral infections. Cerebral hernia is a protrusion of the pia mater or pia mater and a portion of the brain through a hollow in the cranium, in 80% of cases located in the occipital region, i.e. the occipital squama or the foramen magnum. However, it can be present in areas of each cranial bone (frontal, parietal, temporal), as well as the nasal cavity and orbits. In 50% of cases, it coincides with hydrocephalus [25].

Another rare disease, estimated to appear in approx. 0.14 per 1000 births, is occipital horn syndrome. The clinical presentation of this disorder mainly includes bone lesions and deformations, with potential non-specific neurological symptoms of a low intensity [26].

The main limitation of this study was a relatively narrow fetal age group, ranging from the 18th to the 30th week of pregnancy, and a small number of cases, including 37 human fetuses.

## Conclusions

1. The morphometric characteristics of the occipital squama ossification center display no laterality differences.
2. The occipital squama ossification center grows linearly with respect to its vertical diameter, logarithmically with respect to its transverse diameters (supraoccipital and interparietal parts) and projection surface area, and to the quadratic function of age with respect to its volume.
3. The obtained morphometric data of the occipital squama ossification center may be considered as normative for their respective prenatal weeks and may contribute to the estimation of gestational age and the diagnostics of congenital defects.

## Supporting information

S1 Data (XLSX)
